# Melittin Suppresses HIF-1α/VEGF Expression through Inhibition of ERK and mTOR/p70S6K Pathway in Human Cervical Carcinoma Cells

**DOI:** 10.1371/journal.pone.0069380

**Published:** 2013-07-23

**Authors:** Jae-Moon Shin, Yun-Jeong Jeong, Hyun-Ji Cho, Kwan-Kyu Park, Il-Kyung Chung, In-Kyu Lee, Jong-Young Kwak, Hyeun-Wook Chang, Cheorl-Ho Kim, Sung-Kwon Moon, Wun-Jae Kim, Yung-Hyun Choi, Young-Chae Chang

**Affiliations:** 1 Research Institute of Biomedical Engineering and Department of Medicine, Catholic University of Daegu School of Medicine, Daegu, Republic of Korea; 2 Department of Biotechnology, Catholic University of Daegu, Gyeongsan, Republic of Korea; 3 Department of Internal Medicine, Kyungpook National University School of Medicine, Daegu, Republic of Korea; 4 Department of Biochemistry, Dong-A University College of Medicine, Busan, Republic of Korea; 5 College of Pharmacy, Yeungnam University, Gyeongsan, Republic of Korea; 6 Department of Biological Science, Sungkyunkwan University, Suwon, Republic of Korea; 7 Department of Food Science and Technology, Chung-Ang University, Ansung, Republic of Korea; 8 Personalized Tumor Engineering Research Center, Department of Urology, Chungbuk National University, Cheongju, Republic of Korea; 9 Department of Biochemistry, College of Oriental Medicine, Dongeui University, Busan, Republic of Korea; Northwestern University Feinberg School of Medicine, United States of America

## Abstract

**Objective:**

Melittin (MEL), a major component of bee venom, has been associated with various diseases including arthritis, rheumatism and various cancers. In this study, the anti-angiogenic effects of MEL in CaSki cells that were responsive to the epidermal growth factor (EGF) were examined.

**Methodology/Principal Findings:**

MEL decreased the EGF-induced hypoxia-inducible factor-1α (HIF-1α) protein and significantly regulated angiogenesis and tumor progression. We found that inhibition of the HIF-1α protein level is due to the shortened half-life by MEL. Mechanistically, MEL specifically inhibited the EGF-induced HIF-1α expression by suppressing the phosphorylation of ERK, mTOR and p70S6K. It also blocked the EGF-induced DNA binding activity of HIF-1α and the secretion of the vascular endothelial growth factor (VEGF). Furthermore, the chromatin immunoprecipitation (ChIP) assay revealed that MEL reduced the binding of HIF-1α to the VEGF promoter HRE region. The anti-angiogenesis effects of MEL were confirmed through a matrigel plus assay.

**Conclusions:**

MEL specifically suppressed EGF-induced VEGF secretion and new blood vessel formation by inhibiting HIF-1α. These results suggest that MEL may inhibit human cervical cancer progression and angiogenesis by inhibiting HIF-1α and VEGF expression.

## Introduction

Angiogenesis is the physiological process that involves the growth of new blood vessels from pre-existing vessels. It plays an important role in tumor growth, metastasis, and invasion [1], [2]. Most of the factors secreted in cancer cells actually play an important role in generating new blood vessels. Among these factors, the vascular endothelial growth factor (VEGF) is required in the early stages of tumor growth [3]. Many studies have also shown that VEGF is mainly regulated by the hypoxia inducible factor-1 (HIF-1) at the transcriptional level [4].

HIF-1, a transcription factor, regulates many genes involved in adapting to an environment with insufficient oxygen or hypoxia by binding to the hypoxia-response elements (HREs) in the promoter. It is composed of the oxygen-regulated HIF-1α sub-unit and the constitutively expressed HIF-1β subunit [5]. HIF-1α is induced by hypoxia but rapidly decreases when under normoxia. In a normoxic condition, HIF-1α is regulated by the prolyl hydroxylation in the oxygen-dependent degradation domain (ODD). It is modified in proline residues by prolyl hydroxylase; interacts with von Hippel-Lindau (VHL) [6], a recognition component of an E3 ubiquitin-protein ligase [7]; and is targeted for proteosomal degradation [8]. Under hypoxia, stabilized HIF-1α hetero-dimerizes with HIF-1β in the nucleus and activates the transcription of the target genes by binding to their HRE.

In addition, HIF-1α expression is upregulated in response to cytokines and the growth factor. The epidermal growth factor (EGF) also increases the HIF-1α level by activating the epidermal growth factor receptor (EGFR) signaling in a normoxic condition. It has been shown that the EGF-induced mitogen-activated protein (MAP) kinases [9] and phosphatidylinositol 3-kinases (PI3K)/Akt pathways [10] lead to HIF-1α protein synthesis. The MAPK family includes p38, c-Jun N-terminal protein kinase (JNK), and extracellular regulated protein kinase (ERK). Many studies have reported that the PI3K/Akt and MAP kinase pathways regulate VEGF and HIF-1α expression in cancer cells. The mammalian target of rapamycin (mTOR) and p70S6 kinase 1 (p70S6K1), a downstream target of Akt, are likewise implicated in regulating HIF-1α expression [11], [12].

Melittin (MEL), a major component of bee venom, is a 26-amino-acid polypeptide that constitutes 40–60% of dry whole honeybee venom. It has been reported to have multiple effects, such as anti-inflammatory, anti-arthritic, and anti-virus effects in various cell types. It also induces cell cycle arrest, growth inhibition, and apoptosis in various tumor cells [13]. No experiment has yet to demonstrate the molecular mechanisms of the anti-cancer and anti-angiogenesis effects of MEL in cervical cancer cells. In this study, the inhibitory effects of MEL on EGF-induced HIF-1α expression in CaSki cells and the novel mechanisms of the anti-angiogenesis effects of MEL are shown.

## Materials and Methods

### Cells and Materials

Human cervical carcinoma cell lines CaSki cells were obtained from the American Type Culture Collection (USA). Cells were cultured in RPMI 1640 medium supplemented with 1% antibiotic mixture for bacteria and fungi and 10% FBS. These were incubated at 37°C in a humidified atmosphere of 5% CO_2_. All chemicals were obtained from Sigma (St. Louis, MO), unless otherwise indicated.

### Cell Proliferation Assay

To determine effects of MEL on CaSki cell proliferation was evaluated by WST-1 assay. CaSki cells were seeded in a 96-well plate at 2×10^4^ cells/well in RPMI1640 medium and allowed to attach for 24 h. Media was then discarded and replaced with 100 µl MEL of new RPMI1640 medium containing various concentrations of MEL and cultured for 24 h. The cell proliferation reagent WST-1 (Roche applied sicence, Mannheim, Germany) was added to each well. The amount of formazan deposits was quantified according to the supplier’s protocol after 4 h of incubation with WST-1 test solution in 37°C and 5% CO_2_ incubator.

### Western Blot Analysis

Western blotting of all samples was performed as described previously [14] using the indicated primary antibodies and corresponding secondary antibodies specific for whole immunoglobulin from mouse or rabbit (Amersham Biosciences, Buckinghamshire, UK). Immunoreactive proteins were detected using an enhanced chemiluminescence western blotting kit (Roche Diagnosis, Mannheim, Germany) according to the manufacturer’s instructions. Anti-HIF-1α (54/HIF-1 α) and HIF-1β (29) antibody were purchased from BD Transduction Laboratories (San Diego, CA, USA). Specific antibodies for phosphorylation of p70S6 kinase (Thr 421/Ser 424), p44/42 MAP Kinase (Thr 202/Try 204) (E10), Akt (Ser 473) (D9E), JNK (Thr 183/Try 185), mTOR (Ser 2448), and EGFR (Try 1068) (1H12) were purchased from Cell signaling Technology (MA, USA), and β-actin was purchased from Santa Cruz Biotechnology (CA, USA).

### Luciferase Promoter Assay

The ability of MEL to inhibit HIF-1 transcription was determined by the reporter assay dependent on the hypoxia response element (HRE). In brief, at 50–80% confluency, CaSki cells were co-transfected with pGL3-HRE-Luciferase, which contained six copies of HRE derived from the human VEGF gene, and pRL-CMV (Promega, Madison, WI, USA), which encoded Renilla luciferase (Rluc) under the control of a constitutive promoter, using lipofectamine plus reagent (Invitrogen, CA, USA) according to the manufacturer’s instructions.

### Reverse Transcription-polymerase Chain Reaction (RT-PCR)

Total RNA was extracted from cells using Trizol reagent (Invitrogen, Carlsbad, CA, USA). Reverse transcription was carried out using a commercial kit (Superscript II RNase H-reverse transcriptase, Invitrogen, Carlsbad, CA, USA) and total RNA (1 µg) from CaSki cells, according to the manufacturer’s protocol. The sequences of the primers were as follows: for HIF-1α, 5′-CTCAAAGTCGGACAGCCTCA-3′ (sense) and 5′-AATGAGCCACCAGTGTCCAA-3′ (antisense); for VEGF, 5′-CTACCTCCACCATGCCAAGT- 3′ (sense) and 5′-TCTCTCCTATGTGCTGGCCT-3′ (antisense); for GLUT-1, 5′-TTCACTGTCGTGTCGCTGTTT-3′ (sense) and 5′-AGCGCGATGGTCATGAGTAT-3′ (antisense); for β-actin, 5′-GCCATCGTCACCAACTGGGAC-3′ (sense) and 5′-CGATTTCCCGCTCGGCCGTGG-3′ (antisense). PCR products were visualized by 1% agarose gel electrophoresis with ethidium bromide staining.

### Enzyme-linked Immunosorbent Assay (ELISA)

Supernatants at 24 h during stretch were collected. The supernatant of CaSki cell served as a positive control. VEGF enzyme-linked immunosorbent assay was performed with the Quantikine human VEGF kit from R&D Systems (Minneapolis, MN), according to the manufacturer’s instructions. The VEGF standard curve was generated by serial dilution of a stock 2000 pg/ml solution of human VEGF under the same culture media conditions as used for the stretch experiment. The standard curve showed no interference of culture media with the detection of VEGF (the culture media contained no VEGF).

### Chromatin Immunoprecipitation (ChIP) Assay

Chromatin immunoprecipitation was performed as outlined by the commercial assay kit (Upstate Biotechnoloy, NY). DNA binding protein was crosslinked to DNA and lysed in SDS lysis buffer containing protease inhibitor. DNA was sheared to 100–300 bp fragments by sonications with a VC100 sonicator (Sonics & Materials Inc., Danbury) and immunoprecipitated with HIF-1α (H1alpha67) antibody were purchased from (NOVUS Biologicals,Littleton, CO) and anti-rabbit IgG (EMD bioscience, Gibbstown, NJ) overnight at 4°C. A proximal region in the VEGF promoter was amplified form the immunoprecipitated chromatin by polymerase chain reaction (PCR) using the following primer set: sense, 5′-AGACTCCACAGTGCATACGTG-3′ and antisense, 5′-AGTGTGTCCCTCTGACAATG-3′.

### Matrigel Plus Assay

C57BL/6N mice (female, 5-weeks-old) were purchased from Samtako (Osan, Korea) and maintained in pathogen-free conditions. CaSki cells at subconfluence were harvested, washed with PBS, and re-suspended in serum-free medium. Aliquots of cells (3×10^6^) were mixed with 0.5 ml of matrigel in the presence or absence of EGF (500 ng/ml) and MEL (1 and 2 µg/ml). Immediately, the mixture was subcutaneously injected into mice. The mice were sacrificed when tumors were visible, and the matrigel plugs were carefully separated from adjacent tissue and removed. All surgical and experimental procedures used in this study were approved by the Institutional Review Board Committee at Daegu Catholic University Medical Center which conforms to the US National Institutes of Health guidelines for care and use of laboratory animals.

### Transwell Invasion Assay

Matrigel-coated filter inserts that fit into 24-well migration chambers were obtained from Becton-Dickinson (New Jersey, USA). The upper insert of a transwell was coated with 30 µl of a 1∶ 2 mixture of matrigel: PBS. The CaSki cells were plated on the matrigel-coated upper chamber, and the media of presence or absence of drugs added to the upper chamber of the transwell insert. The lower chamber was filled culture medium. Cell in the chamber was incubated for 24 h at 37°C and cells that invaded the lower membrane surface were fixed with methanol and stained with hematoxylin and eosin. The cells that passed through the matrigel and were located on the underside of the filter were counted. Random fields were counted by light microscopy.

### Statistical Analysis

All results are representative of at least three independent experiments done in triplicate; statistical significance between experimental and control values was calculated with using one-way ANOVA test.

## Results

### Melittin (MEL) Inhibited HIF-1α Protein Accumulation Induced by the EGF in the CaSki Cells

**Figure 1:**
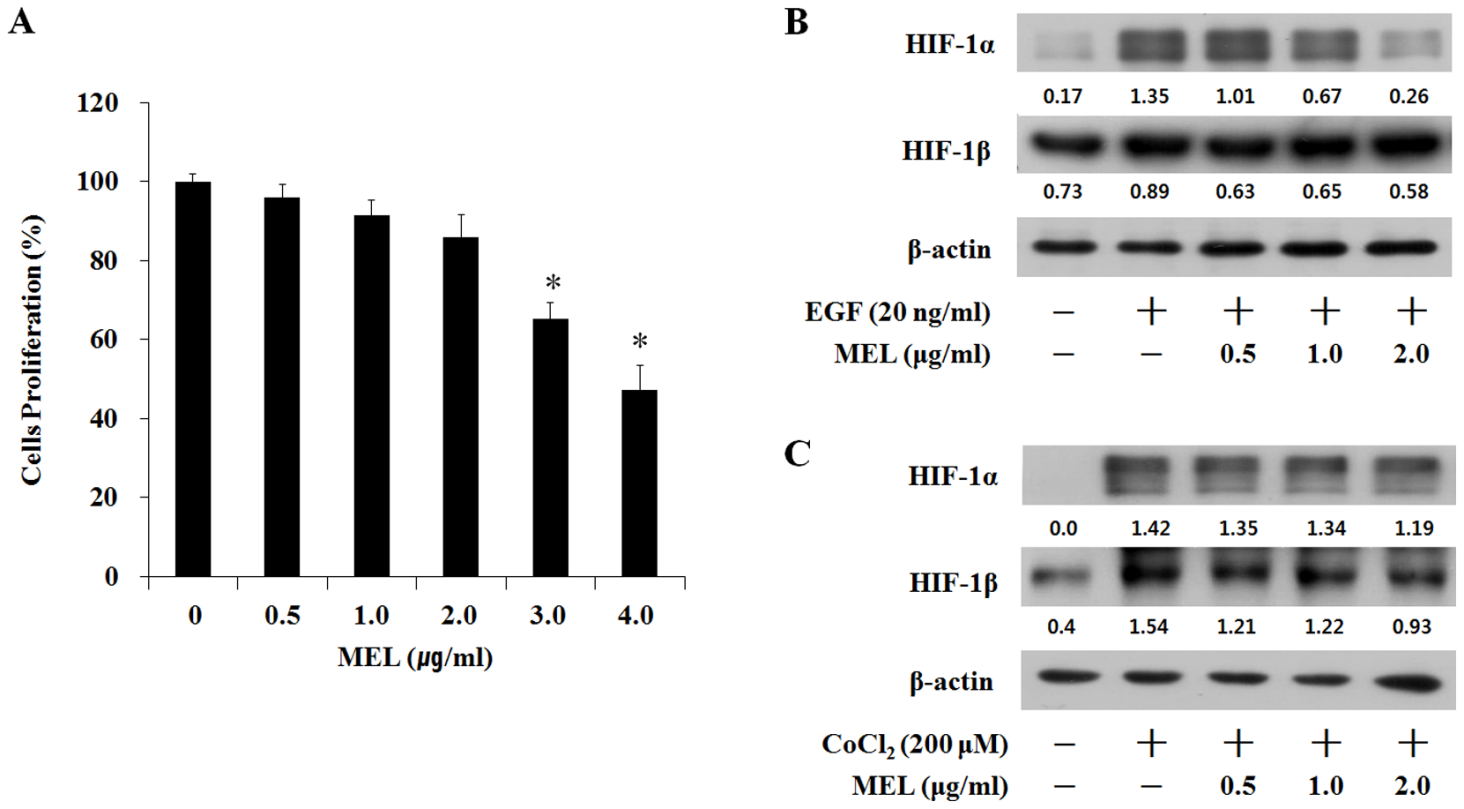
Melittin (MEL) inhibited the HIF-1α protein accumulation induced by the EGF in the CaSki cells. (A) Dose-dependent effect of MEL on the viability of CaSki cells. Cells were treated with the indicated concentrations of MEL for 24 h. Viability was determined by WST-1 assay. Values represent the means ± SD of triplicate assays. *, *p*<0.05 as compared to untreated control. Results were analyzed using one-way ANOVA. (B) Cells were pretreated with the indicated concentrations of MEL for 30 min, and then induced by EGF treatment for 6 h. (C) Cells were pretreated with the indicated concentrations of MEL for 30 min, and then induced by CoCl_2_ treatment for 6 h. Nuclear extracts were subjected to Western blot using antibodies against HIF-1α and HIF-1β.

Before investigating pharmacological potentiality of MEL, its cytotoxic effects in CaSki cells were examined using an MTT assay. The viability of the CaSki cells did not change with 0.5, 1, and 2 µg/ml of MEL, but significantly decreased with 3 and 4 µg/ml of MEL (Fig. 1A). These results show that MEL concentrations of more than 3 µg/ml inhibit the viability of CaSki cells. Therefore, the next experiments were performed in the non-toxic MEL concentrations of 0.5, 1 and 2 µg/ml. We investigated the ability of MEL to modulate the expression of the EGF-induced HIF-1α protein. As shown in Fig. 1B and 1C, EGF (20 ng/ml) and CoCl_2_ (200 µg/ml) treatment significantly increased the HIF-1α protein, but the HIF-1β protein level was only changed by CoCl_2_ in the CaSki cells. The MEL treatment dose-dependently reduced the EGF-induced HIF-1α protein (Fig. 1B), but did not change the CoCl_2_-induced HIF-1α protein expression (Fig. 1C). There results show that the inhibitory effect of MEL on the HIF-1α protein expression involves EGF-specific regulation, and that MEL could also have an inhibitory effect on the angiogenesis.

### Melittin (MEL) Inhibited HIF-1α Expression by Decreasing its Stability

**Figure 2:**
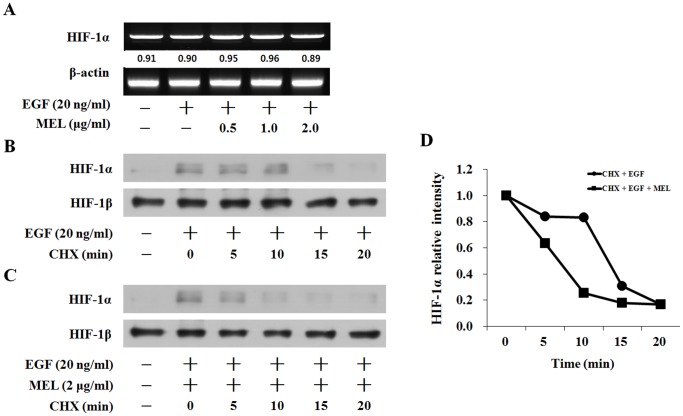
Melittin (MEL) inhibited the HIF-1α expression by decreasing its stability. (A) RT-PCR analysis of HIF-1α mRNA was carried out using total RNA prepared from CaSki cells incubated with EGF treatment for 12 h in the presence or absence of the indicated concentrations of MEL. (B) Cells were pretreated with the indicated EGF for 6 h, and then were treated to CHX time-dependent. (C) Cells were pretreated with the indicated EGF for 6 h. After 6 h, it was cotreated to MEL and CHX (10 µM) time-dependent. (D) Levels of HIF-1α protein were determined by measuring the density of HIF-1α protein band.

To confirm the effect of MEL on the EGF-induced HIF-1α transcriptional activity, RT-PCR was performed. As shown in Fig. 2A, the HIF-1α mRNA expression level could not be induced under the EGF treatment condition, and the MEL treatment did not change the HIF-1α mRNA level under the EGF-induced condition. To confirm the effect of MEL on the EGF-induced HIF-1α protein level, its effect on the stability of the HIF-1α protein was also determined using cycloheximide (CHX) treatment to inhibit new protein synthesis in the cells. The CHX treatment time-dependently reduced the EGF-induced HIF-1α protein level as shown in Fig. 2B. In the MEL and CHX co-treatment case, however, the EGF-induced HIF-1α protein level was quickly reduced (Fig. 2C). The half-life of the MEL-treatment of EGF-induced HIF-1α was shortened by 10 min compared with the CHX-treatment of EGF-induced HIF-1α (Fig. 2D). These results suggest that the inhibition of the HIF-1α protein level is caused by the shortened half-life by MEL.

### Melittin (MEL) Suppressed the EGF-induced HIF-1α Protein Synthesis by Inhibiting the ERK and mTOR/p70S6K Signaling Pathways in the CaSki Cells

**Figure 3:**
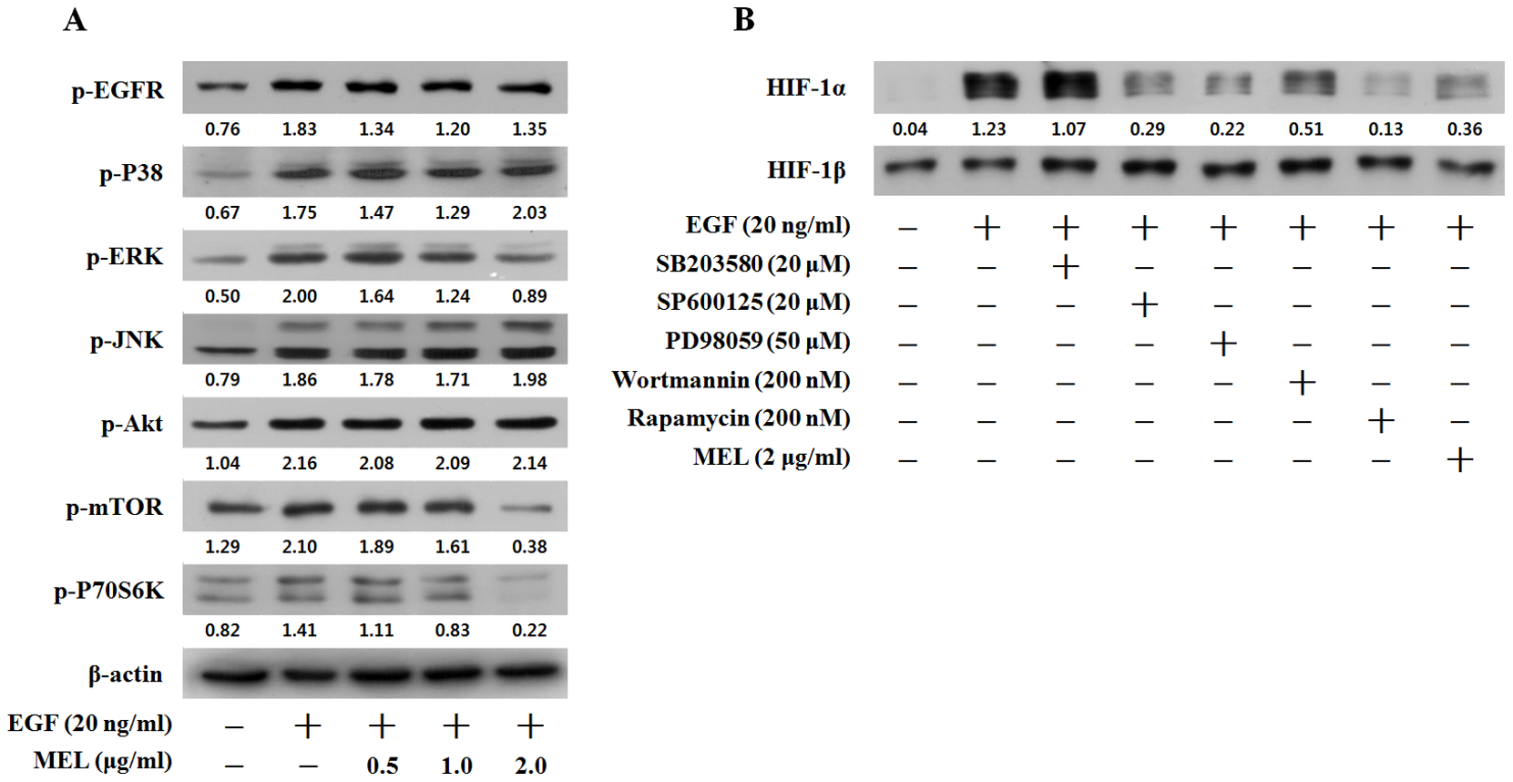
Melittin (MEL) suppressed the EGF-induced HIF-1α protein synthesis by inhibiting the ERK and mTOR/p70S6K signaling pathways in the CaSki cells. (A) Effect of MEL on EGF-induced signaling leading to the expression of HIF-1α in CaSki cells. CaSki cells were pretreated with the indicated concentrations of MEL for 1 h, followed by incubation with EGF for 30 min. The phosphorylated levels of EGFR, P38, ERK, JNK, Akt, mTOR and p70S6K were determined by Western blot analysis. (B) Effects of MEL and inhibitors on EGF-induced expression of HIF-1α in CaSki cells. CaSki cells were pretreated with MEL, SB203580, SP600125, PD98059, wortmannin, rapamycin for 30 min, and then induced by EGF treatment for 6 h. Nuclear extracts were subjected to Western blot using antibodies against HIF-1α or β-actin. Data represents the means ± SD of three independent experiments. *, *p*<0.05 as compared to EGF-treated control. Results were analyzed using one-way ANOVA.

**Figure 4:**
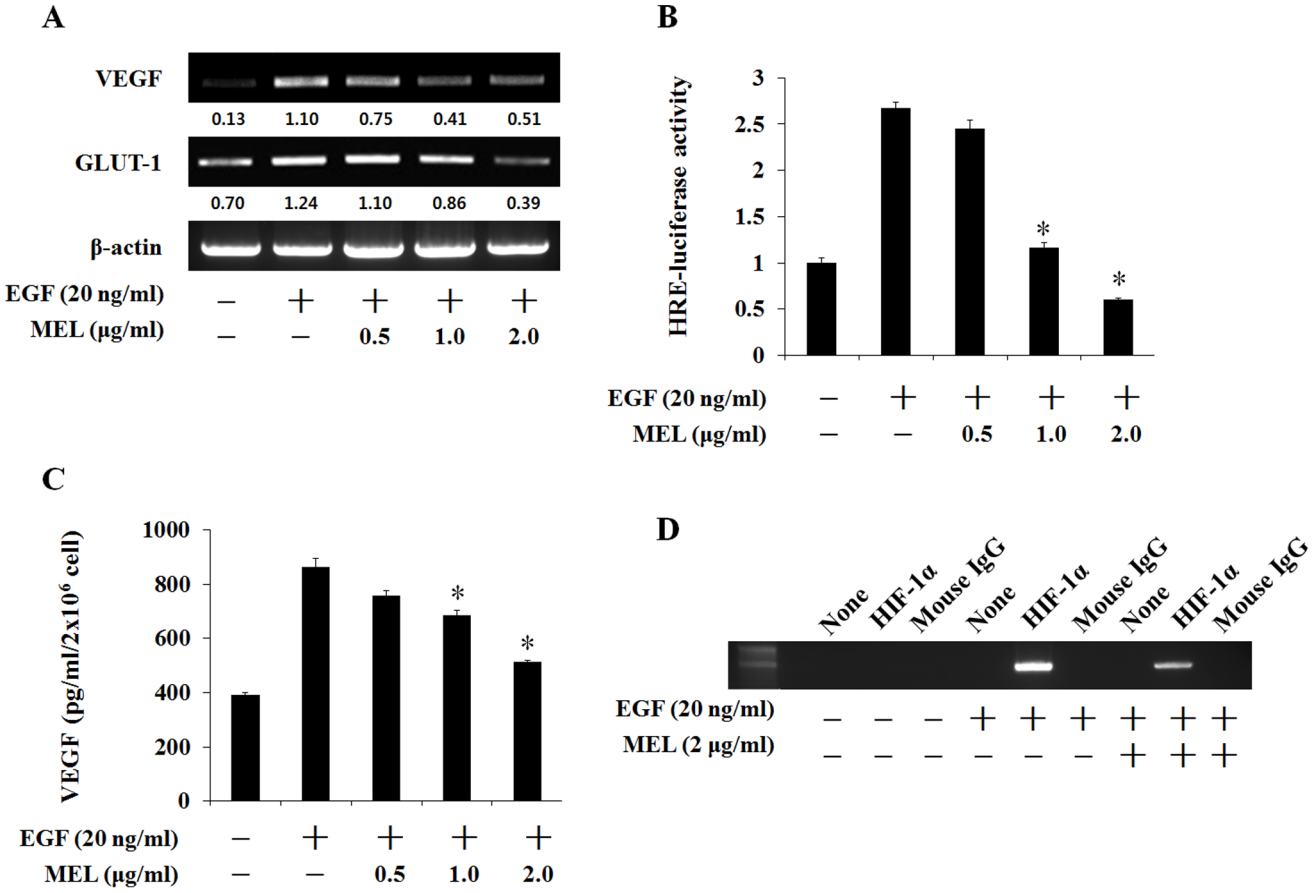
Melittin (MEL) abolished the expression/secretion of the EGF-induced VEGF in the CaSKi cells. (A) RT-PCR analysis of VEGF, and GLUT-1 mRNA was carried out using total RNA prepared from CaSki cells incubated with EGF treatment for 12 h in the presence or absence of the indicated concentrations of MEL. (B) CaSki cells were transiently co-transfected with a reporter gene, pGL3-HRE-Luciferase, and pRL-CMV as a reference. Following incubation for 24 h, the cells were incubated with EGF treatment in the presence or absence of indicated concentration of MEL. (C) VEGF protein expression was evaluated by ELISA in culture supernatant of CaSki cells after incubation under EGF treatment in the presence or absence of the indicated concentrations of MEL. (D) CaSki cells were treated with MEL on EGF-induced for 6 h. The immunoprecipitated DNA with mouse normal IgG and HIF-1α antibody was amplified by PCR analysis for VEF promoter HRE region.

Previous studies showed that the MAPK and Akt/mTOR pathways are involved in the HIF-1α protein induced in cells by EGF treatment [15]–[17]. To determine the mechanism of the HIF-1α regulation by MEL, the MAPK and Akt/mTOR pathways were measured using western blotting. As shown in Fig. 3, the phosphorylation levels of EGFR, p38, ERK, JNK, Akt, mTOR and p70S6 kinase significantly increased after the EGF treatment at a 20 ng/ml concentration, and the phosphorylation levels of ERK, mTOR and p70S6 kinase decreased with a 2 µg/ml concentration of MEL (Fig. 3A). However, the phosphorylation levels of EGFR, p38, JNK and Akt did not change after the MEL treatment. To confirm whether the MAPK, Akt and mTOR pathway activity is required in EGF-induced HIF-1α expression, the CaSki cells were exposed to various kinase inhibitors. As shown in Fig. 4B, SP600125 (20 µM), PD98059 (20 µM), wortmannin (200 nM) and rapamycin (200 nM) decreased the EGF-induced HIF-1α expression by the same degree as MEL did (2 µg/ml) in the CaSki cells. The EGF-induced CaSki cells were also treated with SB203580 (20 µM). In contrast to the aforementioned kinase inhibitors, SB203580 had no effect on the EGF-induced HIF-1α expression in the CaSki cells. SP600125 and wortmannin treatment decreased the EGF-induced HIF-1α protein level. However, MEL, as shown in Fig. 3A, did not affect the phosphorylation of JNK and Akt, indicating that the inhibitory effects of MEL on EGF-induced HIF-1α were not associated with such decrease. These results suggest that MEL suppressed the EGF-induced HIF-1α protein synthesis by inhibiting the ERK and mTOR/p70S6K signaling pathways.

### Melittin(MEL) Abolished the Expression/secretion of the EGF-induced VEGF in the CaSKi Cells

VEGF expression is regulated mainly at the transcriptional level by HIF-1α, an important regulator of angiogenesis [18], [19]. To determine whether the inhibitory effect of MEL on HIF-1α protein expression affects VEGF expression, the VEGF mRNA level was determined using an RT-PCR assay. As shown in Fig. 4A, MEL dramatically and dose-dependently decreased the VEGF mRNA level expression in the EGF-stimulated CaSki cells. In addition, GULT-1, another downstream gene of HIF-1α, decreased with 2 µg/ml of MEL. The HRE-dependent reporter assay was performed to investigate whether these effects of MEL involved the HRE-promoter activity in the EGF-induced CaSki cells. As shown in Fig. 4B, the HRE promoter activity increased 2.5-fold after the EGF (20 ng/ml) treatment, but rapidly decreased with the 1 and 2 µg/ml MEL treatment. The effects of MEL on the secretion of the VEGF protein were further examined via ELISA under an EGF-induced condition in the CaSki cells. The secretion of the VEGF protein selectively increased more than two-fold under the EGF-induced condition (Fig. 4C). The secreted VEGF protein decreased with the 1 and 2 µg/ml MEL treatment in the EGF-induced condition. In addition, a chromatin immunoprecipitation (ChIP) assay was performed. As shown in Fig. 4D, the binding activity of HIF-1α to the VEGF promoter was increased. However, the MEL treatment suppressed binding of HIF-1α to the VEGF promoter region. These results suggest that MEL regulated the VEGF level by inhibiting the HIF-1α.

### Melittin (MEL) Suppressed EGF-induced Migration and Angiogenesis in the CaSki Cells

**Figure 5:**
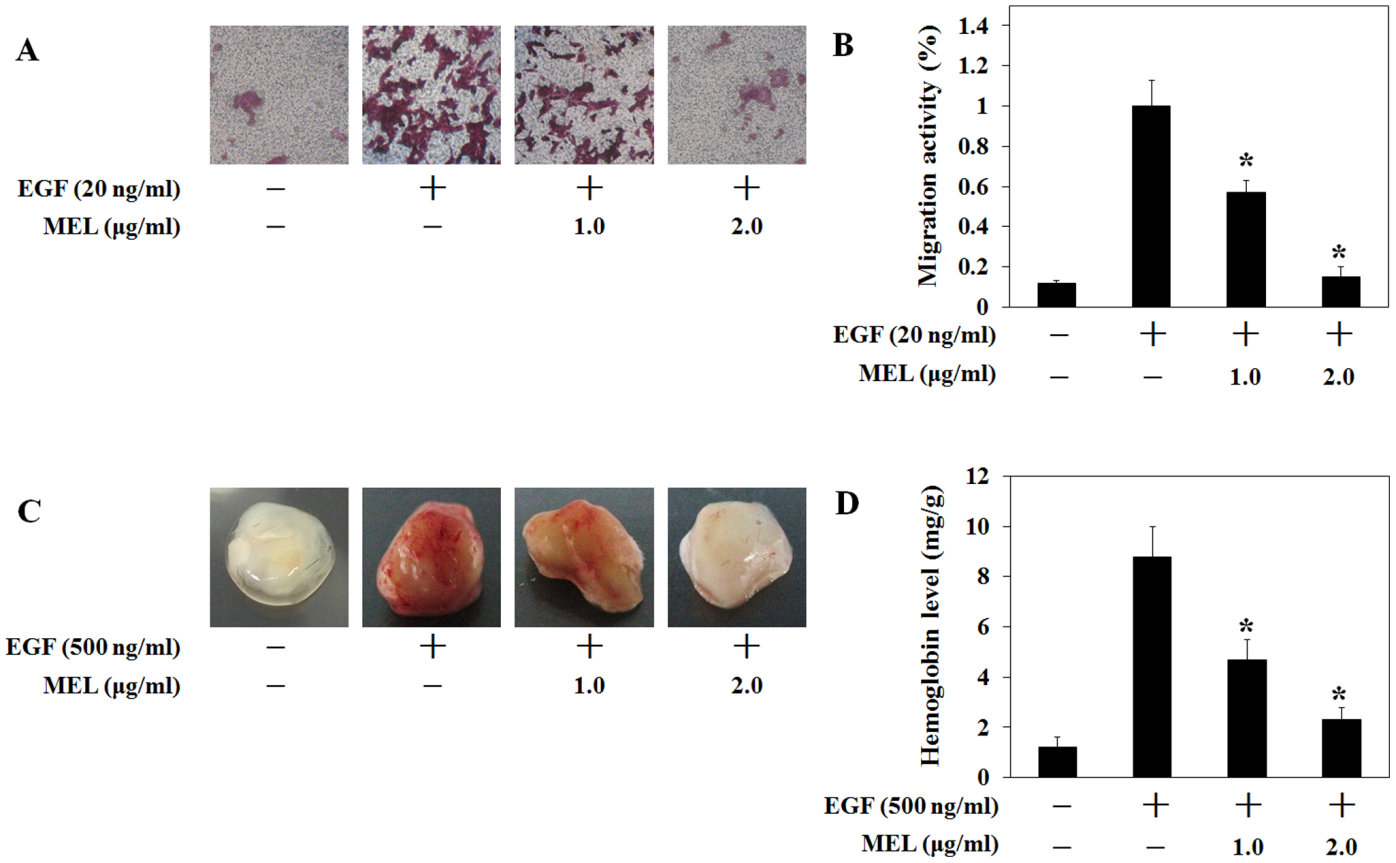
Melittin (MEL) suppressed the EGF-induced migration and angiogenesis in the CaSki cells. (A and B) CaSki 3×10^4^ cells/ml were mixed with 0.5 ml of Matrigel in the presence or absence of EGF (500 ng/ml) and MEL (1 and 2 µg/ml) in vivo. (C and D) Matrigel migration assay was carried out MEL (1 and 2 µg/ml) in the presence of EGF (20 ng/ml). After 24 h incubation, cells bottom side of filter were fixed, stained and counted. Data represents the means ± SD of three independent experiments. *, *p*<0.05 as compared to EGF-treated control. Results were analyzed using one-way ANOVA.

To confirm that MEL decreases the migration of EGF-induced CaSki cells, a transwell invasion assay was performed. As shown in Figs. 5A and 5B, the migration of the CaSki cells increased with the EGF treatment but significantly decreased with the 1 and 2 µg/ml MEL treatment. Also, to further investigate the anti-angiogenesis effects of MEL, an *in vivo* matrigel plug assay in C57BL/6N mice was performed. As shown in Figs. 5C and 5D, the CaSki cells in the presence of EGF induced new blood vessel formation in the plug from the nearby tissues. Although 1 µg/ml of MEL partially inhibited the EGF-induced angiogenesis in the plug, 2 µg/ml of MEL more potently blocked angiogenesis, and the matrigel plugs looked more transparent than with the EGF-induced control (Fig. 5C). These results suggest that MEL blocked the migration of cells as well as new blood vessel formation.

## Discussion

Angiogenesis plays a significant role in the metastasis, invasion and growth of tumors. An increased level of VEGF protein is involved in the angiogenesis and prognosis of cancer, which shows the vital role of VEGF in tumor angiogenesis and development. It has been proposed that a wide variety of cytokines is secreted by cancer cells such as VEGF. VEGF expression is chiefly regulated at the transcriptional level by HIF-1 under a hypoxia and growth factor condition [20], [21]. HIF-1 is composed of HIF-1α and HIF-1β subunits. HIF-1 activates the transcription of many genes that are involved in multiple tumor growth conditions, including angiogenesis, cell survival and invasion [22].

HIF-1α is regulated in cellular responses and plays an important role in angiogenesis and tumor progression [22]. Previous studies have shown that HIF-1α protein expression is induced by hypoxia, CoCl_2_ and EGF [23]. According to a current report, MEL inhibits cell growth and induces apoptosis by inhibiting the MAPK, Akt, and NF-κB pathways in cancer cells [24], [25]. However, MEL has not been reported to have an anti-angiogenesis effect through HIF-1α protein decrease in cancer cells. In this study, the potential anti-angiogenesis effect of MEL was examined by regulating the molecular mechanisms of HIF-1α in CaSki cells. EGF and CoCl_2_ treatment considerably induced HIF-1α protein expression in the CaSki cells (Figs. 1B, and 1C). MEL treatment dramatically reduced the EGF-induced HIF-1α expression (Fig. 1B), but did not change the CoCl_2_-induced HIF-1α expression (Fig. 1C). These results show that MEL reduced the EGF-induced HIF-1α expression.

Earlier studies have confirmed that various small molecules inhibit hypoxia- or growth-factor-induced HIF-1α protein accumulation by stimulating the degradation or decreasing the synthesis rate of the protein [18], [26], [27]. Thus, the inhibitory effects of MEL on EGF-induced HIF-1α protein accumulation were examined using CHX treatment. The EGF-induced HIF-1α protein expression was reduced with CHX treatment (Fig. 2B), but the EGF-induced HIF-1α protein expression was rapidly reduced time-dependently with the MEL and CHX cotreatment (Fig. 2C). The half-life of the EGF-induced HIF-1α was 15 min in the presence of CHX alone in the CaSki cells, and was decreased to 10 min with the MEL treatment. This suggests that the inhibition of the HIF-1α protein level is due to the shortened half-life by MEL (Fig. 2D).

In addition, activated EGFR can regulate HIF-1α protein synthesis and cell proliferation through EGF binding. It has been reported that HIF-1α protein synthesis is needed to regulate the EGF-mediated activation of the PI3K/Akt/mTOR and MAPK pathways by phosphorylating protein translational regulators, including p70S6K [15]–[17]. As such, the pathways are involved in the inhibitory effects of MEL on the EGF-induced HIF-1α in the CaSki cells. It was found that MEL inhibits the expression of the HIF-1α protein synthesis via the ERK, mTOR, and p70S6K pathway (Fig. 3A). Also, PD98059, rapamycin, and MEL treatment considerably reduced the HIF-1α protein expression (Fig. 3B). These results suggest that MEL reduced the ERK-mediated HIF-1α protein expression in the EGF-induced CaSki cells. SP600125 and wortmannin reduced the EGF-induced HIF-1α expression in the CaSki cells, but SB20358 did not change. These results are consistent with previous findings that HIF-1α protein expression was regulated by JNK, Akt, and p38 inhibitors [28], [29]. MEL did not significantly change the phosphorylation levels of Akt and JNK, which suggests that the inhibitory effects of MEL on EGF-induced HIF-1α expression are not relevant to the JNK and Akt signaling pathway.

HIF-1 mainly targets genes such as VEGF and GLUT-1 as well as enzymes of glycolysis [19]. As shown in the results of the experiment on the mRNA of these genes, MEL dramatically reduced the VEGF and GULT-1 mRNA expression in the CaSki cells (Fig. 4A). Moreover, MEL decreased the HRE promoter activity in a manner similar to that with the EGF-induced HIF-1α protein and the VEGF mRNA expression in the CaSki cells. This suggests that the VEGF mRNA level decreased through the inhibition of the HRE promoter activity by MEL (Fig. 4B). Also, the EGF-induced VEGF secretion in the CaSki cells was inhibited by the MEL treatment in the CaSki cells (Fig. 4C), while MEL remarkably inhibited the EGF-induced binding of HIF-1α to the VEGF promoter HRE region (Fig. 4D). These results suggest that MEL specifically suppressed EGF-induced VEGF secretion and HRE promoter activity by inhibiting HIF-1α in the CaSki cells. Consequently, migration of CaSki cells (Fig. 5A) and new blood vessel formation (Fig. 5C) were dramatically suppressed by MEL in the EGF.

To conclude, this study showed that MEL decreases the HIF-1α protein synthesis by inhibiting the ERK, mTOR and p70S6K pathways in an EGF-induced condition. Moreover, MEL showed an anti-angiogenesis effect through decreased VEGF expression by inhibiting the HIF-1α protein. Therefore, this study demonstrated that MEL has a potential anti-angiogenic effect by inhibiting EGF-induced HIF-1α and VEGF protein expression in tumors.
